# Nonadaptive radiation in damselflies

**DOI:** 10.1111/eva.12269

**Published:** 2015-05-27

**Authors:** Maren Wellenreuther, Rosa Ana Sánchez‐Guillén

**Affiliations:** ^1^ Evolutionary Ecology, Biology DepartmentLund UniversityLundSweden; ^2^ Plant and Food Research LimitedNelsonNew Zealand

**Keywords:** adaptive radiation, damselflies, diversification, mechanical isolation, neutral theory, nonadaptive radiation, odonates, sexual selection

## Abstract

Adaptive radiations have long served as living libraries to study the build‐up of species richness; however, they do not provide good models for radiations that exhibit negligible adaptive disparity. Here, we review work on damselflies to argue that nonadaptive mechanisms were predominant in the radiation of this group and have driven species divergence through sexual selection arising from male–female mating interactions. Three damselfly genera (*Calopteryx*,*Enallagma* and *Ischnura*) are highlighted and the extent of (i) adaptive ecological divergence in niche use and (ii) nonadaptive differentiation in characters associated with reproduction (e.g. sexual morphology and behaviours) was evaluated. We demonstrate that species diversification in the genus *Calopteryx* is caused by nonadaptive divergence in coloration and behaviour affecting premating isolation, and structural differentiation in reproductive morphology affecting postmating isolation. Similarly, the vast majority of diversification events in the sister genera *Enallagma* and *Ischnura* are entirely driven by differentiation in genital structures used in species recognition. The finding that closely related species can show negligible ecological differences yet are completely reproductively isolated suggests that the evolution of reproductive isolation can be uncoupled from niche‐based divergent natural selection, challenging traditional niche models of species coexistence.

## Introduction

Radiations are defined as an increase in taxonomic diversity within a rapidly multiplying lineage and can be classified as either adaptive or nonadaptive. The epithet adaptive is used when radiations are driven by ecological diversification that confers individuals an advantage in niche exploitation (Schluter 2000, 2001; Gavrilets and Vose 2005). The term ‘adaptive radiation’ was first applied by the palaeontologist Osborn (1902) to describe parallel adaptations and convergence of species groups. Since then, the concept of adaptive radiations has rapidly increased in popularity and gained widespread recognition during the formulation of the modern synthesis, where they were used as the ultimate showcases of evolution through natural selection (e.g. Huxley 1942).

Despite the long‐standing interest in adaptive radiations, comparatively little attention has been given to nonadaptive radiations, even though the concept dates back to the early 1930s (Wright 1931). Nonadaptive radiations arise through processes that are unrelated to niche exploitation, and thus where reproductive isolation is not linked to the build‐up of ecological niche diversification (Gittenberger 1991; Rundell and Price 2009). Instead, species in nonadaptive radiations diversify through mechanisms that cause modifications to the chromosomal architecture, gene duplication or ploidy levels, or through processes arising from male–female mating interactions, such as sexual selection, sexual conflict and learning (Gittenberger 1991; Rundell and Price 2009). The persistent lack of interest in nonadaptive radiations has limited the number of studies that have explicitly dealt with the concept and to date only a few examples exist (e.g. Cameron et al. 1996; Mendelson and Shaw 2005; Kozak et al. 2006; Comes et al. 2008; Pereira and Wake 2009; Wilke et al. 2010).

One of the best examples comes from Hawaiian *Laupala* crickets, which show no ecologically distinguishable features, are dietary generalists and exhibit little host‐plant dependency, yet species in this genus show the highest speciation rates recorded in arthropods (Mendelson and Shaw 2005). Despite the lack of ecological differentiation, *Laupala* males can be easily differentiated by their distinct courtship songs, strongly indicating that divergence in sexual behaviour has driven the rapid speciation rates in this genus. Furthermore, even though explicit case studies of nonadaptive radiations are rare, a closer inspection of studies on reproductive phenotypes suggests that nonadaptive causes have, at least partially, also been involved in several so‐called ‘adaptive radiations’ (Kaneshiro 1983; Henry 1985; Shaw 1996a,b; Seehausen et al. 1997; Seehausen and Van Alphen 1999). A mutual contribution of both adaptive and nonadaptive processes is to some extent not unexpected, because the dichotomous distinction between the two models is artificial in most cases. The comprehensive work on the African *mbuna* cichlid diversification is a good illustration of how both processes can be implicated in a sequential fashion. First, an ancient adaptive divergence in cichlid jaw morphology allowed species to diversify ecologically and to partition trophic niches, which was subsequently followed by a second bout of species diversification through divergence in male nuptial coloration and associated female preferences for divergent male phenotypes (Danley and Kocher 2001).

In this review, we summarize work on damselflies (Odonata: Zygoptera) to argue that nonadaptive processes appear to be major drivers of species diversification in this group. Damselflies are one of the oldest winged insects that still inhabit the earth, first appearing during the Carboniferous period around 350–300 Mya (Misof et al. 2014), and distinctive features of this group are the ubiquitous reproductive morphologies and behaviours. We have been working extensively on damselflies over the years, both in the field and in the laboratory (see Box 1 for a summary of personal reflections) and focus this review on the genera *Calopteryx*,* Enallagma* and *Ischnura*. We find that ecological niche differentiation between closely related species in these genera is often negligible, commonly leading to sympatric distributions and neutral community dynamics. In stark contrast, reproductive behaviours and associated morphologies appear to be under strong sexual selection and diverge rapidly, indicating that species diversification proceeds principally via male–female interactions. Thus, it appears that the processes leading to nonadaptive diversification in damselflies are mostly driven by sexual selection and only to a minor extent by natural selection, and that the outcome of this nonadaptive diversification process commonly leads to neutral community assemblages. We discuss these results in detail and highlight the implications for traditional niche models of species coexistence.

Box 1 **Personal Reflections**

We have both been interested in science for as long as we can remember. During our early career, we had the fortune of being trained in good institutions alongside some fantastic scientists that helped us learn and grow. While our commitment to science has always been rock‐solid, there have been some unnecessary barriers along the way which took effort to overcome. Some of these have been related to our gender, some of them to our age, and others we are not sure of the causative agents. We would like to highlight two issues that we faced repeatedly that warrant mention, namely (1) unconscious bias against female scientists and (2) battles over research territories and ownership. We have repeatedly been labelled as being ‘too outspoken’, ‘too independent’, ‘too dominant’ or simply just as ‘working too much’. Having that odd little *too* adjective applied to us was an interesting phenomenon and gave us the subtle feeling that we as females were not supposed to show these attributes. When people say ‘you are independent’, it is a compliment. When they say ‘you are too independent’, it is a criticism. Moreover, when I (MW) returned to work part time following the birth of my two children, several academics commented on my ‘too fast’ return to work. My husband received no such comments (at this stage, we were sharing child rearing 50:50). These types of bias are often unconscious, but pernicious. Having the confidence to ignore prejudices has been crucial, as has a strong belief that it's OK not to conform to gender stereotypes. The second major obstacle concerns navigating research territoriality of senior academics over projects that were developed mutually. Such attitudes impede progress in science, and this kind of territorial warfare can greatly harm junior academics that still have an enduring career ahead of them. Claims of seniors to ‘own’ a species or a ‘question’ is something that we both have heard several times. Our advice to other budding scientists is to trust your instincts and to stay calm. Ideally, young academics and their seniors should address issues regarding project ownership before collaboration is started. In our case, we realised too late that conditions of research ownership were placed on us. Science can be overly competitive and some academics simply take advantage of junior collaborators without sufficient regard for their career. While obstacles along our careers have led to unpleasant periods, we both feel very fortunate at the current point in our careers and can say that the positive experiences by far outweigh the negative. A career in science is an extreme job without the financial benefits or job securities provided by other career paths, but a career in science provides much freedom and can be extremely satisfying. Our advice to young academics is to seek a mentor or sponsor that can share some of their experiences and importantly, can also do a bit of trumpet blowing on your behalf. Our plea to more experienced scientists is to reach out to younger colleagues who are trying to find their way.

## Criteria for genera selection and phylogenetic relationships

The suborder Zygoptera consists of almost 3000 species distributed in over 300 genera; however, the work has been biased towards only a few key genera. Studies on niche diversification have primarily concentrated on adults of the genus *Calopteryx* (Wellenreuther et al. 2010a; Wellenreuther et al. 2012), *Enallagma* (McPeek et al. 1996; McPeek and Peckarsky 1998; Brown et al. 2000; Turgeon and McPeek 2002; Siepielski et al. 2010) and *Ischnura* (Wellenreuther et al. 2011; Sánchez‐Guillén et al. 2012), while a lot less is known about larval ecology. Comparatively, more attention has been given to the role of nonecological selection on reproductive traits (see Box 2 for a summary of damselfly reproductive biology), including in *Argia* (Paulson 1974), *Calopteryx* (Svensson et al. 2007; Svensson et al. 2010, 2014; Tynkkynen et al. 2008b; Wellenreuther et al. 2010a,b), *Enallagma* (Robertson and Patterson 1982; McPeek 1995; McPeek et al. 2008, 2009, 2011), *Ischnura* (Paulson 1974; Monetti et al. 2002; Sánchez‐Guillén et al. 2005, 2011a; Sánchez‐Guillén et al. 2012, 2013a,c) and *Nehalenia* (Van Gossum et al. 2007). Given that the aim of this study was to examine the relative importance of ecological and nonecological forces in fuelling species diversity in damselflies, only those genera for which sufficient information was available for both categories were selected. This criterion was fulfilled for *Calopteryx, Enallagma* and *Ischnura*.

Box 2 **Reproductive biology**

**Figure 1:**
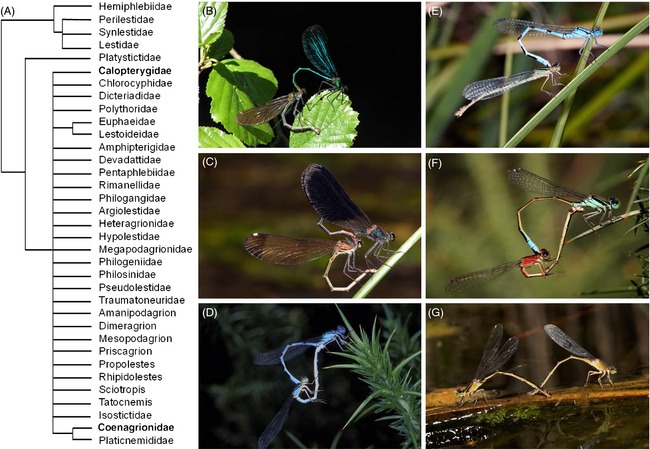
Phylogenetic relationships of damselfly families and some representative species. (A) depicts a phylogenetic tree of 35 zygopteran families (redrawn from Dijkstra and Kalkman 2012) to indicate the phylogenetic position of the family Calopterygidae and Coenagrionidae. (B) shows a *Calopteryx virgo* male and female in tandem position. (C) shows a male *Calopteryx haemorrhoidalis* transferring sperm to a female. (D) shows a *Enallagma cyathigerum* male and androchrome female in the wheel position. (E) shows an *Enallagma cyathigerum* male and gynochrome female in the wheel position. (F) shows a male and a gynochrome (*aurantiaca*) female *Ischnura graellsii* in the wheel position. (G) shows a *Ischnura graellsii* gynochrome (*infuscans*) females during oviposition. Photograph B was taken by Maren Wellenreuther and photographs E–G were taken by Adolfo Cordero Rivera.

Odonata are unique among insects in affording two separate morphological contact points to copulate (Paulson 1974). First, when a male finds a suitable female (this step includes male choice, Fig 1B), he must grasp the female by her mesostigmal plates (secondary sexual genitalia located in the prothorax) with his abdominal appendages (secondary sexual genitalia: cerci and paraprocts) to achieve the tandem position (Fig. 1C). Odonata males have the primary (testes) and secondary genitalics (penis) disconnected, thus males have to transfer sperm from the testes to the penis prior to copulation (Leonard and Córdoba‐Aguilar 2010). Second, the female must accept copulation (this step includes female choice and species recognition) by bending her abdomen to allow contact between both primary genitalia and to form the wheel position (Fig. 1D,E). The mating wheel allows copulation (the intromission of the penis to the vagina). Copulation takes place in two stages (Miller and Miller 1981). During the first stage (of variable duration), the male carries out a series of abdominal movements to remove sperm from previous matings, although a stimulatory species‐specific function is also likely. During the second stage (of fairly constant duration), the sperm is transferred. In some species, copulation is followed by a third stage (mate guarding) during which the male retains the female in the wheel or tandem position (Fig. 1F) to avoid re‐mating.

Reproductive isolation in damselflies is seldom caused by a single isolating barrier, but more commonly by multiple isolating mechanisms (Sánchez‐Guillén et al. 2012, 2014a,b). Premating reproductive barriers in damselflies include habitat, temporal, sexual and mechanical isolation. Of these, temporal and habitat isolation are caused by ecological divergence, whereas sexual isolation (also called behavioural isolation) and mechanical isolation evolve through male–female mating interactions. Postmating reproductive barriers prevent the formation of offspring, or reduce hybrid offspring viability and fertility. Factors leading to postmating isolation include prezygotic barriers (reduced sperm insemination and sperm removal rate, failure to stimulate female oviposition, reduced fecundity and sterility) and postzygotic barriers (hybrid viability, hybrid sterility and reduced hybrid vigour).

The phylogenetic relationships of most damselfly groups have not been resolved in detail, and thus, the speed of lineage diversification and the exact routes of species splitting are challenging to reconstruct. Here, we summarize what is known about the phylogenetic history and position of the three focal genera. The Holarctic genus *Calopteryx* (Fig. 1B,C) originated around 35 Mya (Dumont et al. 2005) and consists of 26 geographically widespread species in North America, Asia and Europe (Misof et al. 2000). Large parts of their vast Holarctic territory have been subjected to Pleistocene glaciations, which has compressed species ranges into isolated pockets during pleniglacials (Weekers et al. 2001). Subsequent range expansions into the formerly glaciated areas occurred during interglacials, and expansions are in some cases ongoing (e.g. Wellenreuther et al. 2012). The Eurasian *Calopteryx* group (*C. haemorrhoidalis*,* C. splendens*,* C. virgo* and *C. xanthostoma*) is monophyletic (Weekers et al. 2001) and began to radiate around 6.2 Mya, and the first product of this radiation, around 5.3 Mya, was the *C. virgo* group (*C. virgo* and *C. haemorrhoidalis*), while the *C. splenden*s group (*C. splendens* and *C. xanthostoma*) appeared after 3.7 Mya (Dumont et al. 2005).

*Enallagma* and *Ischnura* both belong to the family Coenagrionidae and are closely related. *Proischnura*,* Coenagriocnemis*,* Africallagma*,* Aciagrion* and *Azuragrion* together form the sister clade to *Enallagma,* and this group forms the sister genus to *Ischnura* (S. Bybee, personal communication). Recent reconstructions of the phylogenetic relationships suggest that *Enallagma* damselflies (Fig. 1D) are monophyletic (Seth Bybee, personal communication) and are present on all continents except Australia and Antarctica. The *Enallagma* genus started to radiate around 10–15 Mya (Dijkstra and Kalkman 2012) and encompasses around 70 species. Their global distribution shows two centres of diversification: North America and sub‐Saharan Africa, with a few scattered species around the Asian and Palaearctic region (Brown et al. 2000). For instance, only four species have been recorded from the Palearctic region (*E. circulatum*,* E. cyathigerum*,* E. deserti* and *E. risi*) and only *E. cyathigerum* is found in Europe. The Nearctic group, in contrast, contains 38 species and is one of the most speciose odonate groups. The majority of these species are found in North America, making it the most diverse damselfly group in that region. Based on its biogeography, the radiation of the North American *Enallagma* species includes two monophyletic clades: the southern ‘*hageni*’ and the northern ‘*carunculatum*’ clades (Brown et al. 2000). Data indicate that about half of all extant *Enallagma* species have arisen sometime within the last 250 000 years from these two radiating lineages, and most species arose within the last ~15 000 years (Brown et al. 2000; Turgeon and McPeek 2002; Turgeon et al. 2005).

*Ischnura* (Fig. 1E,F) species ages range between 25 and 45 Mya (Bechly 1998), and encompasses around 70 species that are distributed on all continents, with the exception of Antarctica (Dijkstra and Kalkman 2012). Phylogenetic relationships of the 15 North American ischnurans show a recent diversification along a latitudinal gradient (Chippindale et al. 1999). The North American group is divided into two main clades. One monophyletic clade including seven taxa (*I. damula, I. demorsa, I. denticollis, I. perparva I. posita posita*,* I. posita atezca* and *I. verticalis*) and a clade including three species (*I. erratica*,* I. cervula* and *I. gemina*). The remaining North American species *I. barberi*,* I. kellicotti*,* I. hastata*,* I. prognata* and *I. ramburii* are thought to represent much earlier divergences in the group (Chippindale et al. 1999). Recent phylogenetic work centred on the Eurasian ischnurans included 14 Eurasian, four North American, two African and two Australian species, and obtained phylogenetic patterns consistent with a recent diversification in this group (Dumont 2013). Three main clades were resolved, namely the Nearctic ‘*hastata’*, the Eurasian *‘pumilio’* and the Palearctic ‘*elegans’* clade (Dumont 2013). The Eurasian ‘*pumilio’* clade was determined to be closest to the Nearctic ‘*hastata’* clade and the Palearctic ‘*elegans’* clade showed signs of a recent radiation centred around the Mediterranean basin. Unfortunately, the young age of the Palaearctic ‘*elegans’* clade has hampered a detailed phylogenetic reconstruction (Dijkstra and Kalkman 2012; Dumont 2013; Sánchez‐Guillén et al. 2014b).

## Evidence for adaptive ecological niche diversification

### Niche conservatism in *Calopteryx*

*Calopteryx* are territorial riverine species that require abundant vegetation for oviposition and hunting (Córdoba‐Aguilar and Cordero‐Rivera 2005). Species ranges commonly overlap near the centre of the distribution, while regions along the still expanding and trailing range margins overlap little or not at all, creating a mosaic of sympatric and allopatric populations (Dijkstra and Lewington 2006; Dijkstra and Kalkman 2012). For example, bioclimatic and environmental niche modelling suggests that differences in *C. splendens* and *C. virgo* species ranges are mostly related to interspecific differences in physiological optima for temperature and precipitation levels (Wellenreuther et al. 2012) (Table 1). However, apart from interspecific differences in physiological tolerances, overall ecological divergence between the species is negligible, and for most other ecological traits, a high degree of niche conservatism is apparent (Wellenreuther et al. 2012), leading to extensive range overlap across the majority of their distribution. Fine scale overlap is also pronounced, and sympatric individuals can frequently be observed to hunt and mate within less than a metre of each other (M. Wellenreuther, personal observation). Fine scale overlap is further supported by work on thermal partitioning (Svensson 2012) and temporal partitioning (M. Wellenreuther, unpublished data), with both species dwelling in almost indistinguishable niches. Finally, gene flow estimates of *C. splendens* populations in southern Sweden are pronounced, suggesting that populations are highly interconnected (Svensson et al. 2014), which may contribute to the low differentiation in ecology. Consistent with high gene flow are the low to moderate *F*_ST_ values of this species (Svensson et al. 2004: *F*_ST_ = 0.05; Chaput‐Bardy et al. 2008: *F*_ST_ = 0.14). Field surveys of *C. haemorrhoidalis*,* C. splendens* and *C. virgo* in Italy and *C. haemorrhoidalis, C. virgo* and *C. xanthostoma* in Spain report of many sympatric areas coupled with significant interspecific overlap in temporal activities (Dijkstra and Lewington 2006). High interspecific habitat overlap was also found between the North American *C. aequabilis* and *C. maculata* during an extensive population survey across the north‐eastern United States and south‐eastern Canada (Waage 1975). The timing of reproduction of the two latter species is also almost synchronous in sympatry (Cameron et al. 1996). While these studies suggest that interspecific overlap in *Calopteryx* spp. is frequent, we would like to highlight that allopatric localities occur and together with the sympatric sites form a microgeographic mosaic of allopatric and sympatric populations that are often only separated by a few kilometres.

**Table 1:** Summary of the evidence that the damselfly genera belonging to *Calopteryx*,*Enallagma* and *Ischnura* have (A) diversified adaptively in ecological niche use and (B) nonadaptively in traits associated reproduction. Unknown denotes topics that have not been explored in these genera

	Genus *Calopteryx*		Genus *Enallagma*		Genus *Ischnura*	
**(A) Evidence for adaptive ecological niche diversification**						
Niche divergence	Small	Wellenreuther et al. (2012)	Minimal	McPeek and Peckarsky (1998) and Siepielski et al. (2010)	Unknown	
Genetic differentiation at the species level	Low differentiation	Svensson et al. (2004) and Chaput‐Bardy et al. (2008)	Low differentiation	Turgeon et al. (2005)	Low differentiation	Wellenreuther et al. (2011), Takahashi et al. (2013) and R. Sánchez‐Guillén, unpublished data
Ecological displacement	Small, 3 species co‐occur in Europe	Dijkstra and Lewington (2006)	Minimal, 12 in North America	Bourret et al. (2012)	Small, 5 in middle Asia	Borisov (2006)
Divergence in the timing of reproduction in sympatry	No, synchronous	Cameron et al. (1996)	No, synchronous	Bourret et al. (2012)	No, synchronous	Borisov (2006) and Sánchez‐Guillén et al. (2005)
**(B) Evidence for nonadaptive diversification in reproduction**						
Visual mate recognition	Yes: Wing melanization and male displays	Svensson et al. (2004, 2007, 2014)	(No) Random mating	Turgeon and McPeek (2002) and Fincke et al. (2007)	(No) Random mating	Sánchez‐Guillén et al. (2012, 2014b)
Mechanical Isolation: precopulatory species recognition	No	Lorenzo‐Carballa et al. (2014)	Yes: strong key and lock mechanisms	Paulson (1974), Robertson and Patterson (1982) and Fincke et al. (2007)	Yes: strong key and lock mechanisms	Sánchez‐Guillén et al. (2012, 2013c)
Postmating morphologies involved in sperm displacement	Yes: male genitalia	Waage (1979a,b, 1986)	Unknown		Unknown	Sánchez‐Guillén et al. (2012)
Gametic isolation	Unknown		Unknown		Yes: lower F_1_, F_2_ and backcrosses fitness	Sánchez‐Guillén et al. (2012)

### Neutral assemblage structure in *Enallagma*

*Enallagma* are nonterritorial damselflies commonly found in the littoral zone near standing or, occasionally, near slow‐flowing water (McPeek and Brown 2000; Siepielski et al. 2011). Phylogenetic studies on the North American representatives using morphological characters and mitochondrial DNA data indicate that fish lakes represent the ancestral habitat of this group and that at least two separate lineages of *Enallagma* (four species) have subsequently invaded dragonfly‐dominated lakes. Ample evidence indicates that this habitat shift was the result of three independent habitat shifts possibly linked to rapid evolutionary changes in morphological, physiological and behavioural traits related to swimming performance (McPeek 1995; Stoks et al. 2003). While niche divergence is implicated in the diversification of four species, the ancestral species in fish dominated lakes are ecological equivalents (Siepielski et al. 2010) (Table 1). This equivalency occurs even though species commonly overlap. Indeed, up to 12 species can be seen to co‐occur at a lake side by side, despite species having coincident or overlapping flight and mating seasons. Elaborate field experiments to investigate demographic factors governing species coexistence found that stabilizing effects (*sensu* Chesson 2000) facilitate coexistence of different sympatric damselfly genera in North America (*Enallagma, Ischnura* and *Lestes*), by causing genera to be limited by different ecological factors (e.g. resources, predators, disease) (McPeek and Peckarsky 1998; Siepielski et al. 2010). Consistent with a role of stabilizing effects in regulating different genera, the abundance of adult and larvae from each genus (sum of individuals of all species of each genus) covaries along environmental gradients (Siepielski et al. 2011). In contrast, species abundance is uncorrelated with environmental gradients, and experimental evidence indicates that equalizing effects regulate *per capita* mortality and growth rates (Siepielski et al. 2010). As a consequence, species assemblage structure conforms to random expectations with ecological factors only regulating the summed total abundance of all species, but not the abundance of individual species (termed a zero‐sum interaction). For this reason, species’ relative abundances on both local and regional scales are not directly affected by local environmental conditions, and hence, species numbers undergo a random walk due to ecological drift (Hubbell 2001). The ultimate outcome of this ecological neutrality is the extinction of all species save one without the continual input of new species or immigration of individuals from the source populations on the outside (Hubbell 2001).

### Conserved ranges and niche overlap in *Ischnura*

*Ischnura* is the most cosmopolitan genus of the family Coenagrionidae, and like *Enallagma*, all species are nonterritorial and show a preference for standing water (Sánchez‐Guillén et al. 2014b). *Ischnura* species are generalist predators and display mostly conserved allopatric ranges as seen in the case of the Palaearctic species in the ‘*elegans’* clade*,* including *I. fountaineae* and the *I. elegans*‐like species *I. elegans*,* I. genei*,* I. graellsii* and *I. saharensis*. Despite their overall conserved distribution, many species are sympatric over reduced parts of their range and, within this range, overlap is mediated by fine scale niche preferences. For example, *I. graellsii*,* I. genei* and *I. saharensis* show preferences for standing and running water with vegetation, while *I. fountaineae* prefers springs and rivers with little vegetation, and *I. elegans* prefers a variety of eutrophic standing waters (Dijkstra and Lewington 2006). Surveys in Middle Asia documented that up to five of the seven species co‐occur (*I. fountaineae*,* I. elegans*,* I. evansi*,* I. forcipata* and *I. pumilio*) (Borisov 2006), and in Morocco, three (*I. fountaineae*,* I. pumilio* and *I. saharensis*) of the four species can be found at the same locality (Jacquemin et al. 1999). Moreover, the three Iberian ischnurans (*I. elegans*,* I. graellsii* and *I. pumilio*) also frequently appear at the same location (R. Sánchez‐Guillén, personal observation) and show overlapping phenological patterns (Sánchez‐Guillén et al. 2012, 2014b). Three large sympatric regions of *I. elegans* and *I. graellsii* exist along the Iberian coastal Peninsula, though overlap within these regions is reduced by *I. elegans* preferring coastal and *I. graellsii* inland habitats (Sánchez‐Guillén et al. 2012). Molecular work across 22 European *I. elegans* populations demonstrates low genetic differentiation (mean *F*_ST_ = 0.06), presumably because of efficient dispersal (Wellenreuther et al. 2011) (Table 1). Similar levels of genetic differentiation were detected across 30 populations of *I. senegalensis* in Japan (*F*_ST_ = 0.10), another wide ranging species of the Palearctic ‘*elegans’* clade (Takahashi et al. 2013). Even species with more restricted distributions show similar levels of genetic differentiation (data derived from four populations of each species covering their distribution: *I. graellsii F*_ST_ = 0.03; *I. genei F*_ST_ = 0.13; and *I. saharensis F*_ST_ = 0.09, R. Sánchez‐Guillén, unpublished data). The molecular data corroborate the idea that *Ischnura* spp. are efficient dispersers, and for example, *Ischnura* is frequently the only zygopteran on many oceanic islands, such as the Azores, Galapagos and some Asian islands. The high dispersal ability of ischnurans presumably dilutes ecological differentiation among populations.

## Evidence for nonadaptive diversification in reproduction

### Morphological divergence and learned mate preferences in *Calopteryx*

*Calopteryx* males are territorial and engage in vigorous male–male competition over oviposition territories along the water (Waage 1973). When a female approaches a territory, she is courted with elaborate wing displays by some of the males in the vicinity. During courtship, males present their melanic and sexually dimorphic wing coloration (Fig. 1B,C) prominently to females, and female choice of suitable mating partners is largely based on this coloration (Córdoba‐Aguilar 2002; Córdoba‐Aguilar et al. 2003; Svensson et al. 2004, 2014). The extent of male wing melanization is used in both intrasexual selection (Córdoba‐Aguilar 2002; Svensson et al. 2004, 2007; Svensson et al. 2014; Córdoba‐Aguilar and Cordero‐Rivera 2005) and in interspecific species recognition (Waage 1975, 1979a,b; Tynkkynen et al. 2004, 2005; Mullen and Andrés 2007; Svensson et al. 2007). The dual use of wing melanization as a trait for intrasexual selection and for recognizing heterospecifics can push colour traits in opposite directions and consequently interfere with one of its functions. For example, large male wing patches are preferred by females of *C. splendens*, but wing colour is also a species recognition trait to distinguish this species form the almost fully melanized congener *C. virgo* (Svensson et al. 2004, 2007, 2014) (Table 1). Thus, the extent of realized wing melanization is strongly dependent on the local circumstances, and dynamically reflects the dynamics of intra‐and intersexual selection pressures. Similarly, classic work on the North American *C. maculata* and *C. aequabilis* demonstrated wing pattern displacement and increased mate discrimination in sympatry and has since served as one of the few classic examples of speciation via reinforcement outside of *Drosophila* (Waage 1975, 1979a,b). Recent ecological and molecular work on these species confirms that sympatric populations are the result of recent secondary contact, as predicted under a model of speciation via reinforcement. However, the rapid evolution of wing colour in sympatry seems to be better explained by selection against wasting mating effort and/or interspecific aggression resulting from a ‘noisy neighbour’ signalling environment (Mullen and Andrés 2007). Phylogenetic comparative work on *Calopteryx* spp. colour indicates that clear wings represent the ancestral state, and therefore, sexually dimorphic pigmentation is a derived character (Svensson and Waller 2013). This study also reported a link between wing colour and elevated speciation and extinction rates, implying that selection on pigmentation traits may be causal in the splitting of species (Svensson and Waller 2013).

In contrast to the exuberant colour displays of males in this genus, male reproductive abdominal appendages are strikingly similar. As a result, interspecific copulations are easily achieved once a tandem has been formed (Lorenzo‐Carballa et al. 2014), suggesting that hybridization may be widespread. Indeed, interspecific tandems and copulations are recurrent in the field (Keränen et al. 2013; M. Wellenreuther, unpublished data), and both morphological (De Marchi 1990; Dumont et al. 1993) and molecular analyses (Tynkkynen et al. 2008a,b; Keränen et al. 2013; Lorenzo‐Carballa et al. 2014) confirm the presence of hybrids. The morphological work of Dumont et al. reported hybridization between *C. splendens* and *C. xanthostoma* (Dumont et al. 1993), while molecular data have confirmed that hybridization between *C. splendens* and *C. virgo* and between *C. splendens* and *C. haemorrhoidalis* is reciprocal, and that both F_1_ hybrids and backcrosses are produced (albeit at low densities for the latter pair) (Tynkkynen et al. 2008a,b; Keränen et al. 2013; Lorenzo‐Carballa et al. 2014).

Despite the limited divergence in male reproductive appendages, postmating morphologies used in sperm displacement are highly differentiated (Waage 1979a,b, 1986). In fact, *Calopteryx* served as a model group for pioneering studies on the mechanics of sperm removal (e.g. Waage 1979a,b, 1986). Males can be categorized into three groups based on their sperm removal tactics: (i) males that gain physical access to the spermathecae, (ii) males that cannot physically remove sperm from the spermathecae, presumably because the spermathecal lumen is too narrow to allow the entry of the male genitalia and (iii) males that elicit sperm ejection from the spermathecae via female sensory stimulation (Waage 1979a,b, 2004; Siva‐Jothy and Hooper 1995; Cordero and Andrés 2002; Cordero Rivera et al. 2004; Tsuchiya and Hayashi 2008). Remarkable variation in sperm removal mechanisms has been described, even among closely related species (i.e. *C. haemorrhoidalis*,* C. splendens* and *C. virgo*, Cordero Rivera et al. 2004). For example, comparative work on allopatric Spanish and Italian populations of *C*. *haemorrhoidalis* observed that male morphology has diverged functionally. In Spain, males reportedly empty the spermathecae by stimulating females, whereas in Italy, males remove sperm physically from the spermathecae (Cordero Rivera et al. 2004). Furthermore, phenotypic differentiation in genitalic traits was much greater compared to differentiation based on seven other morphological traits, consistent with the idea that postmating selection has been an important mechanism in the diversification of this group (Cordero Rivera et al. 2004).

In the last years, evidence has also accumulated that *Calopteryx* exhibit learned mate behaviours and plastic mate preferences. For example, naïve female *C. splendens* can rapidly learn to distinguish between con‐ and heterospecific males based on the amount of wing melanin (Svensson et al. 2007, 2010). Evidence that learning of heterospecific phenotypes is involved in sexual isolation between *Calopteryx* spp. was determined experimentally by presenting *C. splendens* females to heterospecific *C. virgo* males from allopatric and sympatric areas (Wellenreuther et al. 2010b). In sympatry, *C. virgo* could clearly distinguish between con‐ and heterospecific females, but in allopatry, this ability was significantly decreased, leading to a greater likelihood of heterospecific interactions. While loss of premating species recognition in *C. virgo* males could have been partly caused by genetic drift, repeated experiments on various ontogenetic stages indicate that learning is the dominant force (Svensson et al. 2007; Wellenreuther et al. 2010b). Most recently, a study on *C. splendens* and *C. virgo* investigated how sex differences and plasticity in mate preferences can affect population divergence in the face of gene flow (Svensson et al. 2014). By combining field and molecular data, it could be demonstrated that male species recognition is fixed at emergence, whereas females can swiftly learn to distinguish conspecific from heterospecifics. This greater plasticity may allow females to respond more efficiently to local changes in the frequency of heterospecifics and therefore may protect the population from species mixing (Svensson et al. 2014).

### Lock and key reproductive isolation in *Enallagma*

Damselflies in the genus *Enallagma* lack precopulatory courtship, and behavioural as well as visual species recognition is poorly developed, leading to almost random mating attempts among congeneric species. Field observations of *E. ebrium* and *E. hageni* showed, for example, that males lack an innate mate preference and fail to distinguish between their phenotypically and genetically similar females (Turgeon and McPeek 2002; Fincke et al. 2007). This lack of a pre‐existing sensory bias results in naïve *E. ebrium* males engaging sexually with both female morphs of *E. hageni* as often as they do to their own females (Fincke et al. 2007; see Box 3 about a summary of colour polymorphisms). The resulting frequency‐dependent reproductive interference between these species may have played an unsuspected role in accelerating genetic differentiation in the early phases of nonecological speciation, with reinforcement further consolidating reproductive isolation between lineages (Bourret et al. 2012). While precopulatory selection is minimal or absent in this genus, field studies have shown that species recognition mechanisms act once physical contact has been established. Specifically, when a male tries to initiate mating, the female immediately tests whether the male cerci fit with the shape of their mesostigmal plates (see Box 2). These structures are akin to a lock‐and‐key mechanism, and thus may allow each sex to efficiently discriminate between species (Paulson 1974; Robertson and Patterson 1982; Fincke et al. 2007). Field work has observed that even though intraspecific differentiation in male cerci is minimal to absent, cerci shape is highly differentiated between species, thus ruling out geographic factors or drift as the main cause of reproductive divergence (McPeek et al. 2011). Detailed morphological investigations of reproductive structures in this genus have been conducted with the help of scanning electron microscopy. Images were collected for six species (*E*. *glaucum, E. rotundipenne, E. sapphirina, E. nigridorsum, E. sinuatum* and *E. subfurcatum),* and for each male, the inferior abdominal appendages (paraprocts) that press on the female prothorax and the superior cerci that press on the female mesostigmal plates were measured. The results showed that the superior male appendages differed markedly and were congruent with the distribution of the female mesostigmal sensilla (Robertson and Patterson 1982). Subsequent work including all except two *Enallagma* species (*E. desertii* and *E. truncatum*) was able to detect correlated evolution between male and female secondary genitalics (male cerci and female mesostigmal plates), further highlighting the role of these structures in species recognition (McPeek et al. 2008, 2009). Specifically, co‐occurring species from the ecologically and phenotypically similar clades ‘*hageni’* and ‘*carunculatum’* differ markedly in their genital structure so that individuals can be grouped into species based on the male and female secondary sexual characters alone (McPeek et al. 2011) (Table 1). The hypothesis that incompatibility in secondary genital structures is the main force preventing hybridization was also verified experimentally by altering cerci shapes. When conspecific males had their cerci shapes modified and were subsequently presented to females, females immediately rejected conspecific males (Robertson and Patterson 1982). Thus, the morphologies of secondary male and female structures appear to be critical for mate recognition and acceptance, underscoring that evolution of mechanical isolation has been fundamental in the radiation of this group. The finding that mechanical isolation is, however, not complete between all species indicates past asymmetric hybridization, which is consistent with the finding that species are genetically compatible and, therefore, could hybridize (Turgeon et al. 2005).

Box 3 **Sex‐limited colour polymorphism**

Sex‐limited colour polymorphisms exemplify extreme morphological diversity within a sex and are generally rare. Odonates are an exception and contain >100 species with female‐limited polymorphism (Fincke et al. 2005) and a handful of species with male‐limited polymorphism (Van Gossum et al. 2008). Mapping and crossing studies demonstrate that colour polymorphisms are heritable, and that the genetic basis is due to simple Mendelian inheritance of 1–2 loci, or alternatively, a set of tightly linked loci (e.g. Tsubaki 2003; Sánchez‐Guillén et al. 2005). The majority of female‐limited colour polymorphisms consist of two or more colour morphs, of which one typically resembles conspecific males in coloration and behaviour (termed androchrome morph), while the other(s) females show less conspicuous coloration (termed gynochrome morphs). The prevalence of female‐limited polymorphism in this group is thought to be an evolutionary response by females to sexual conflict over optimal mating rates, where females benefit from lower mating rates than males, and where density‐ and frequency‐dependent male mating harassment is common (Sánchez‐Guillén et al. 2011b; Sánchez‐Guillén et al. 2013b). In contrast to the variable number of female colour morphs, male colour polymorphism always consists of two morphs. In *Megalagrion sp*., for example, males display either an orange or blue coloration, which is in striking contrast to the green coloration of females (Polhemus and Asquith 1996). The evolution of male‐limited colour polymorphism has been explained in terms of alternative male mating tactics (Van Gossum et al. 2008), and indeed, colour polymorphic males often include a territorial fighter and a ‘sneaky’ male, the latter resembling conspecific females in phenotype and succeeds by intercepting females during mating.

Theoretical arguments (Gray and McKinnon 2007; Wellenreuther et al. 2014) and empirical data (Hugall and Stuart‐Fox 2012) suggest that colour polymorphism can accelerate speciation rates. Specifically, environment‐contingent sexual selection and selection arising from sensory bias can cause divergence between populations, with the balance between selection and gene flow influencing the likelihood of speciation versus polymorphism persistence (Gray and McKinnon 2007). Of these, a link between a mating preference and colour appears to be a particularly straightforward way to induce population divergence (Gray and McKinnon 2007), but contemporary examples are scare. A possible case where polymorphism has led to a recent speciation comes from the ecologically and morphologically similar sister species *Palpleura lucia* and *P. porta*. These species are completely reproductively isolated from one another, and although females are indistinguishable morphologically, they commonly coexist in sympatry. The only documented difference between these species is male wing patterning, indicating a male colour polymorphism predated speciation (Mitchelu and Samways 2005; Van Gossum et al. 2008). Evidence that colour polymorphism can fuel species diversity comes also from phylogenetic comparative studies. In the family Coenagrionidae, the most specious genera show some of the highest frequencies of colour polymorphism (e.g. *Argia*,* Coenagrion*,* Enallagma* and *Ischnura*), and the presence of monandry/polyandry seems to be correlated. Monandrous species are mainly monomorphic, while polyandrous species are typically polymorphic (Robinson and Allgeyer 1996). We are currently working towards understanding the genetic basis of colour in *Ischnura* spp. to study the micro‐ and macroevolutionary processes that have generated and maintain colour differences in this fascinating group.

### Rapid evolution of secondary sexual appendages in *Ischnura*

**Figure 2:**
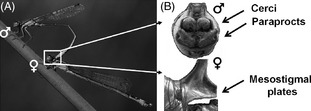
Reproductive morphology of damselflies. (A) shows first mating contact point (tandem) of the secondary sexual traits in *Ischnura elegans*. (B) shows the male and female secondary genitalics which consist of male abdominal appendages on the 10th abdominal segment (cerci and paraprocts) and the female prothorax mesostigmal plates. Photographs were taken by Adolfo Cordero Rivera.

Similar to *Enallagma* damselflies, ischnuran damselflies are nonterritorial and consequently show little courtship behaviour and visual displays. A significant body of research on *Ischnura* damselflies (Fig. 1D,E) has focussed on sexual conflict and the ubiquitous female limited colour polymorphism (Box 3). Studies examining sexual behaviours have found no evidence for interspecific differentiation, even between closely related European species such as *Ischnura elegans*,* I. genei* and *I. graellsii,* leading to almost random premating interactions (Sánchez‐Guillén et al. 2012, 2014b). In the two North American species *I. denticollis* and *I. gemina* (which belong to different clades), males clearly prefer conspecific over heterospecific females (Sánchez‐Guillén et al. 2014a), indicating some degree of precopulatory recognition and selection. In contrast to the limited extent of behavioural divergence, morphological differentiation in primary and secondary genitalia is pronounced and commonly leads to asymmetric mechanical incompatibilities (Sánchez‐Guillén et al. 2012, 2014b) (Table 1). As in *Enallagma,* species recognition takes place during mating via tactile interactions between male abdominal appendages and female mesostigmal plates (Fig. 2B) and the female can then accept or refuse to cooperate with the male. If the female accepts copulation, postmating structural isolation due to incompatibility between primary genitalics can additionally prevent hybrid formation. This postmating barrier is either caused by aberrant morphology of the primary genitalics or by inappropriate male movements, after which females prematurely interrupt copulation, refuse oviposition (Sánchez‐Guillén et al. 2012, 2013c) or expel heterospecific sperm (RAG‐S, personal observation).

*Ischnura elegans* ranges from Ireland to the Mediterranean and Japan, whereas its sister species *I. graellsii* (Fig. 1F) has a more restricted distribution in the western Mediterranean area (Iberia and Maghreb). Anthropogenic changes in recent years have allowed *I. elegans* to extent into central and western Spain where it now forms large secondary contact zones with *I. graellsii*. In these newly created sympatric populations, the species frequently hybridize, showing a pattern of introgressive hybridization of genes from *I. graellsii* into *I. elegans* (Monetti et al. 2002; Sánchez‐Guillén et al. 2005; Sánchez‐Guillén et al. 2011a). In Iberia, *I. elegans* and *I. graellsii* show strong asymmetry in premating mechanical isolation consistent with a modified version of the Kaneshiro's model where *I. graellsii* is the derivative species because of its restricted range and *I. elegans* the progenitor species (Sánchez‐Guillén et al. 2012). This pattern can explain the high premating mechanical isolation when *I. graellsii* males mate with *I. elegans* females (93% of heterospecific matings are impeded), and the almost complete absence of isolation when *I. elegans* males mate with *I. graellsii* females (only 13% impediment). More recently, we have extended our studies to two other Mediterranean *I. elegans*‐like species, namely *I. genei* and *I. saharensis*. *Ischnura genei* is restricted to the Tyrrhenian Islands and partially overlaps with *I. elegans* (Boudot et al. 2009), while *I. saharensis* is restricted to Morocco and partially overlaps with *I. graellsii*. Both species show extensive hybridization throughout the area of contact: *I. genei* with *I. elegans* and *I. graellsii* with *I. saharensis* (Sánchez‐Guillén et al. 2014b). We also tested premating (temporal, sexual and mechanical) and postmating (oviposition success, fecundity and fertility) isolation between two novel species combinations, namely (i) between *I. genei* and *I. elegans*; (ii) between *I. genei* and *I. graellsii*. The findings corroborated that mechanical isolation is pervasive in all species combinations, impeding between 60% and 95% of matings (Sánchez‐Guillén et al. 2014b). The most detailed work on the relative importance of different reproductive barriers in damselflies was carried out by measuring the strength of 19 reproductive barriers between *I. elegans* and *I. graellsii*, including for the first time postzygotic mechanisms (F_1_‐hybrid fitness, F_1_‐hybrid fertility, F_2_‐hybrid sterility and F_2_‐hybrid vigour). We found that postzygotic barriers contributed much less than premating and postmating prezygotic barriers to the total reproductive isolation underscoring that the evolution of premating barriers is key factor in the diversification of *Ischnura* spp. (Sánchez‐Guillén et al. 2012).

**Figure 3:**
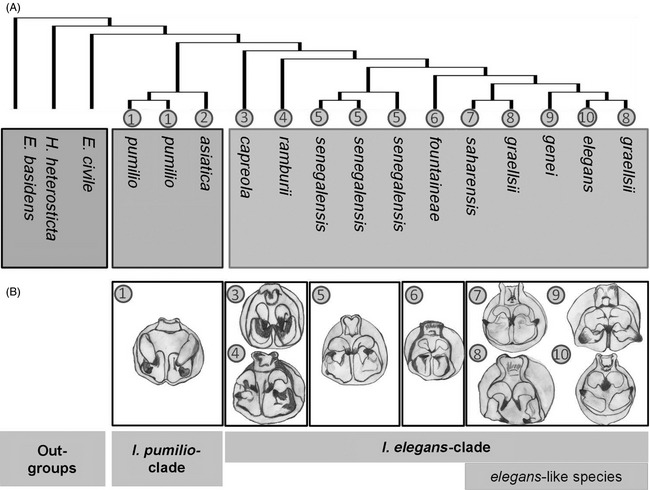
Phylogenetic relationships of ischnuran species and male abdominal appendages. (A) Maximum likelihood (RAxML) tree (redrawn fr om Sánchez‐Guillén et al. 2014b) derived from 669 informative positions of the cytochrome oxidase II and the cytochrome b mitochondrial regions. (B) Posterior view of the male abdominal anal appendages of 10 *ischnuran* species. Pictures were taken with the LAS software (Leica Microsystems) and then redrawn by hand.

The accumulating evidence suggests that the diversification of ischnuran species likely proceeded in allopatry or parapatry via divergence in secondary sexual male abdominal appendages, which either impeded copulation or affected female tactile preferences (Sánchez‐Guillén et al. 2012, 2014b). Figure 3 shows phylogenetic relationships (Fig. 3A) and male abdominal morphological structures (Fig. 3B) of 10 ischnuran species representing the *I. pumilio* and the *I. elegans* clades*. Ischnura pumilio* cerci have triangular plates, while cerci in the *I. elegans* clade species have broad and rounded plates with a strong internal tooth (Fig. 3B). The four *I. elegans‐like* species occur mainly allopatrically and are morphologically very similar except for their genitalia, the latter which can be used to reliably group individuals into species based on the prothoracic tubercles and male abdominal appendages: *I. elegans* exhibits parallel cerci, *I. graellsii* curved cerci and *I. genei* and *I. saharensis* crossed cerci (Fig. 3B) (Sánchez‐Guillén et al. 2014a).

## Discussion

Here, we reviewed the relative importance of ecological and nonecological processes in the radiation of the three damselfly genera *Calopteryx*,* Enallagma* and *Ischnura*. In all three cases, the degree of interspecific ecological niche diversification was minimal relative to the pronounced diversity in characters involved in reproduction. Specifically, species differentiation in the territorial genus *Calopteryx* appears to have been driven predominantly by divergence in wing melanization important in intrasexual selection and interspecific species recognition. In the nonterritorial genera *Enallagma* and *Ischnura*, however, reproductive isolation was mostly achieved through morphological alterations of mating structures involved in tactile species recognition. We note that the finding that closely related species often differ in reproductive traits is in itself no rigorous proof that sexual selection was causative in the diversification of these genera, because interspecific differences could have arisen after reproductive isolation was already accomplished. However, similar arguments could also be made about ecological differences, as these too could have emerged after or intensified after species splitting was completed. While it will undoubtedly be impossible to reject either scenario outright, the majority of studies on the three genera support the notion that sexual interactions have been fundamental in the diversification of this group, and that reproductive barriers for the majority of species arose largely independent of ecological differences (Gittenberger 1991; Rundell and Price 2009). Some exceptions exist. For example, speciation in some *Enallagma* species appears to have been triggered by niche shifts from lakes dominated by fish predators to lakes dominated by dragonflies as top predators (McPeek et al. 1996). It seems likely that the complex reproductive morphologies and wide diversity in mating behaviours of damselflies makes them particularly amendable to evolve in response to sexual interactions, as the range of reproductive complexities provides ample material for selection to act on.

### Nonadaptive radiations: uncoupling of ecology and reproductive isolation

Nonadaptive radiations driven by sexual selection result in new species that are ecologically similar to their progenitors, thus the increase in species diversity is not accompanied by ecological niche diversification. The same pattern is also expected for nonadaptive speciation events triggered by autotetraploidy (e.g. Ramsey and Schemske 1998) or chromosomal rearrangements (e.g. King 1995; Rieseberg 2001). Nonadaptive radiations caused by allotetraploidy and hybridization can present a potential exception to the uncoupling of ecology and reproductive isolation, however, even if ecological differentiation is involved in these case, it is typically nonadaptive and arbitrary, as the generation of diversity is unrelated to the available niches in the system (Rieseberg 1997). Therefore, in nonadaptive radiations, the increase in species diversity is not accompanied by an increase in functional diversity, but rather species are added to already existing functional groups. A direct but underappreciated consequence of the uncoupling between ecology and reproduction is that the overall potential for diversification is ultimately higher and the resulting species diversity can hence exceed the number of available niche spaces in the environment. It should be noted, however, that environmental resource limitation can still have a controlling effect in this scenario, but that instead of a species carrying capacity, the total (and combined) number of individuals from all species form the currency that need to be considered. A second consequence of the uncoupling between ecology and reproductive isolation is that the evolutionary age of species derived through nonadaptive processes may be on average shorter than those that have come about through adaptive processes (McPeek and Peckarsky 1998; Siepielski et al. 2010). This is because the ecological similarity of species in nonadaptive radiations gives no species a competitive edge, and it is thus the frequency and density of individuals in the whole assemblage that is limited, but not that of a single species. Consequently, species may be either slowly driven to extinction as their relative abundances vary until only one species remains or is maintained in a local area by dispersal from other areas (Hubbell 2001; McPeek et al. 2008). An inevitable consequence of the minor niche differences is that species from nonadaptive radiations may be particularly prone to go extinct, as these weakly ecologically differentiated species will easily be outcompeted (McPeek and Brown 2000; McPeek et al. 2008; Siepielski et al. 2010). In environments characterized by long stable periods interrupted by short‐term fluctuations, the net changes to species diversity may thus be zero, and thus, lineages derived via nonecological processes may show an overall higher clade volatility (Rosenblum et al. 2012).

Conclusions Ecological niche differentiation has long been the main force used to explain biodiversity, and the limiting similarity theorem (*sensu* Hutchinson 1959) has been ingrained in our ecological thinking as a universal rule. It is therefore not surprising that ecological differences among co‐occurring taxa are often invoked as an explanation for the maintenance of species richness. This has given rise to numerous empirical studies and theoretical treatments of fitness trade‐offs between traits affecting the demographic performances of species along environmental gradients. These studies have undoubtedly been important in showing that a large quantity of species can coexist in sympatry through ecological niche partitioning, but for an even larger portion of species, this assumption is simply taken at face value without the conduction of rigorous experimental tests. It is clear that adaptive radiations provide us with fascinating living libraries to study phenotypic evolution and central evolutionary processes. However, there is a need to recognize that many similar species frequently co‐occur in nature and we think that this observation implies that adaptive processes should not necessarily be applied as the null model for radiations. Rather than dismissing close species similarity as being due to nonequilibrium situations, we should instead give it a second thought and test this assumption. There are a number of testable predictions to evaluate whether nonadaptive sexual processes have contributed to the evolution of species diversity, and we will list four of these here: (i) the community dynamics depend on the total number of individuals in an assemblage, but not the number of species *per se*, so that the removal of one species should have little effect on con‐and heterospecific interactions as long as the total number of individuals remains constant; (ii) the extent of genetic divergence strongly correlates with the degree of reproductive isolation; (iii) species recognition is almost entirely based on lock‐and‐key mechanisms of genitalia; and (iv) gene flow between individuals is strongly linked with sexual morphology, but shows little relationship with environmental factors. When conducting proper tests, we may find that some species are ecologically neutral and that nonequilibrium dynamics may in fact be prevalent in groups (Hubbell 2001). While examples of nonadaptive radiations are scarce (e.g. Gittenberger 1991), the lack of empirical evidence should not be interpreted as being synonymous with a lack of importance. Indeed, it seems likely that once we start to question some of our basic assumptions regarding the need for ecological dissimilarity, we may find that many more candidates exist. The minor niche differentiation of damselflies challenges traditional niche divergence models of species coexistence and the large interspecific differences in reproductive characters points towards sexual interactions as a diversifying force. We suggest that future studies should question the underlying null hypothesis of their models and recognize that assemblages may have evolved in response to the dynamic interplay of the dual action of adaptive and nonadaptive forces to create species diversity.
